# Mitochondrial Dynamics in Cancer and Neurodegenerative and Neuroinflammatory Diseases

**DOI:** 10.1155/2012/729290

**Published:** 2012-06-27

**Authors:** Mauro Corrado, Luca Scorrano, Silvia Campello

**Affiliations:** ^1^University of Geneva, 1211 Geneva, Switzerland; ^2^Venetian Institute of Molecular Medicine, 35129 Padova, Italy; ^3^IRCCS Fondazione Santa Lucia, 00143 Rome, Italy

## Abstract

Mitochondria are key organelles in the cell, hosting essential functions, from biosynthetic and metabolic pathways, to oxidative phosphorylation and ATP production, from calcium buffering to red-ox homeostasis and apoptotic signalling pathways. Mitochondria are also dynamic organelles, continuously fusing and dividing, and their localization, size and trafficking are finely regulated. Moreover, in recent decades, alterations in mitochondrial function and dynamics have been implicated in an increasing number of diseases. In this review, we focus on the relationship clarified hitherto between mitochondrial dynamics and cancer, neurodegenerative and neuroinflammatory diseases.

## 1. Introduction

In eukaryotic cells, the role of mitochondria is pivotal both in providing essential molecules and signals for life and in amplifying signals of death. In regard to the cell life, mitochondria produce most of the ATP necessary to the cell through oxidative phosphorylation, and they are involved, among the others, in TCA cycle, fatty acid metabolism, hemesynthesis, and gluconeogenesis. As regards the cell death, mitochondria are involved in Ca^2+^ and red-ox homeostasis, which are dysregulated during cell death, and they release proapoptotic proteins, such as cytochrome *c*, SMAC/DIABLO, AIF, Endo G, and Omi/HTRA2, after mitochondrial membrane permeabilization and cristae remodeling [1–3]. 

**Figure 1:**
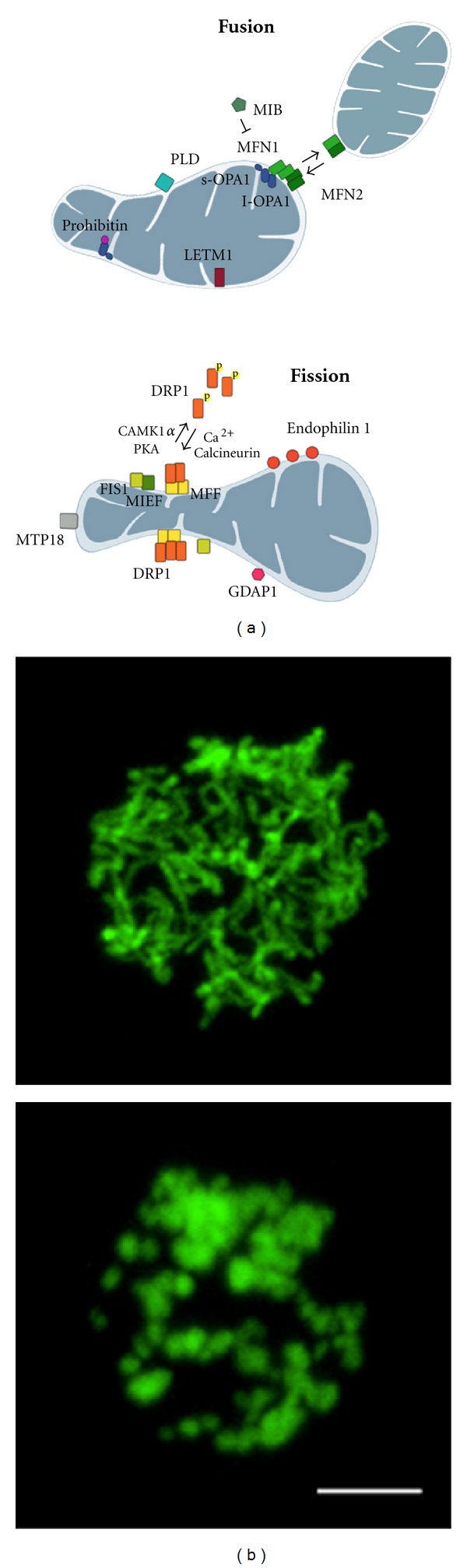
The dynamic nature of mitochondrial shape. (a) Main proteins involved in mitochondrial shape changes are depicted. In fused unopposed condition, DRP1 is phosphorylated and sequestered in the cytoplasm. Once dephosphorylated, it is recruited to the OMM where it oligomerizes and interacts with FIS1, MFF, or MIEF inducing constriction of membranes and, eventually, fission of mitochondria. MFNs homo- and heterooligomerization on the OMM and oligomerization between long and short isoform of Opa1 on the IMM control fusion of mitochondrial membranes. Additional proteins affecting mitochondrial shape are also shown. (b) Mitochondrial morphology in Jurkat cells overexpressing yellow fluorescent protein targeted to mitochondria. The upper panel shows a network of elongated and interconnected mitochondria. In the lower panel, mitochondria appear fragmented (Scale bar: 5 *μ*m).

Moreover, mitochondria are highly dynamic organelles that can fuse and divide, forming an interconnected network or fragmented units inside the cell, according to different stimuli impinging on the fusion/fission machinery, represented by the mitochondria shaping proteins: MFN1, MFN2, OPA1, regulators of fusion, and DRP1, FIS1, MFF, and MIEF1, which modulate fission [4] (see Figure 1(a)).

### 1.1. Mitochondrial Fusion

MFN1 and MFN2, two dynamin-related GTPases, are the main regulators of mitochondrial fusion at the level of outer mitochondrial membrane (OMM). They can interact, forming homo- and heterodimers; after conformational changes led by the hydrolysis of GTP, they force the OMM to fuse [5]. Interestingly, MFN2 is also responsible for ER/mitochondria tethering with an important implication in Ca^2+^ homeostasis and signalling [6]. OPA1, another dynamin-related GTPase, is located in the inner mitochondrial membrane (IMM) where, together with MFN1, it plays a role in controlling fusion at this level [7]. OPA1 has also a role in controlling cell-death; in fact, heterocomplexes between proteolytic processed or unprocessed forms of OPA1 regulate the width of cristae junctions and the subsequent release of cytochrome *c*, which then interacts with APAF1 and caspase 9 forming the apoptosome, whose activation results in the amplification of cell death signals [8, 9]. Other proteins have been linked to mitochondrial fusion, such as LETM1 [10], the Phospholipase D (PLD) [11], and Prohibitins (Phb) [12], the latter necessary for OPA1 processing.

In summary, the pleiotropic mitofusins (MFN1 and MFN2) and OPA1 are the main regulators of the mitochondria fusion machinery. Although steps forward have been made, some points of this mechanism still remain to be clarified; in particular, how the IMM fuses and how its fusion is coordinated with events of the OMM fusion. 

### 1.2. Mitochondrial Fission

The fission machinery is based on DRP1, FIS1, MFF, and MIEF1. DRP1 is a large GTPase protein [13], which in conditions of unopposed fusion is cytosolic and after dephosphorylation by calcineurin is recruited on mitochondria [14], where it oligomerizes and interacts with its putative adaptors on the OMM (FIS1, MFF, and MIEF1), forming ring-shaped structures and inducing mitochondrial constriction and fission [15]. Endophilin B1 [16], MTP18 [17], MIB [18], and GDAP1 [19] have been described, moreover, among the fission components.

The scenario of mitochondrial fission is becoming more complex, taking into account the different roles proposed for MFF, MIEF, and FIS1. MFF has recently been shown to be an adaptor of DRP1 on the OMM [20], whereas the binding of MIEF to DRP1 on the OMM inhibits the GTPase function of DRP1 and its profission activity [21]. On the other hand, FIS1, that initially was considered the only DRP1 receptor at the OMM, has now been proposed to exert its profission role by interacting and sequestering MIEF, thus allowing DRP1 to mediate the constriction and fission of the membrane [21].

It has also been suggested that mitochondrial fission events predominantly occur at the contact sites between ER and mitochondria, specifically where some ER tubules cross over and wrap around mitochondria. Interestingly, DRP1 and MFF localize at these contact sites [22]. New studies that will address the biochemical mechanism, by which the ER participates in mitochondrial division, placing this observation in the overall picture of mitochondrial division, will be of great interest in the field.

In summary, the fission machinery depends on the activation and translocation of DRP1 to the OMM, where it interacts with some other components, not clearly defined, that take part to the modulation of mitochondrial fragmentation. 

### 1.3. More than Just Morphology: The Intimate Connection with Physiological Functions

Changes in mitochondrial morphology have been related to alterations in mitochondrial function, transport, location, and quality control. Mitochondrial damage, for example, induces fission, which, in turn, allows mitochondria to be engulfed by the autophagosomes and then degraded [23].

Autophagy represents a cellular self-degradation process involved in the degradation of bulk cytoplasmic components, proteins, or entire organelles in basal or nutrient depleted conditions. This process is also described as macroautophagy and is different from the selective autophagy. The latter is responsible for selective degradation of damaged and dysfunctional organelles, and, thus, it represents the quality control system for mitochondria [23]. In case of damage, mitochondria undergo fragmentation, and PARKIN is recruited to the organelles in a PINK1-dependent manner, allowing their engulfment into the autophagosome and their selective degradation (mitophagy) [24, 25]. On the other hand, when macroautophagy is induced, mitochondria elongate, being so spared by autophagosomes to ensure the major energy supply required by the cell in starving condition [26]. Besides their role in mitochondrial quality control, PINK1 and PARKIN have also a role in regulating mitochondrial dynamics. Moreover, BNIP3, a BH3-only member of the Bcl-2 family, enlarges the number of proteins crosstalking between autophagy and mitochondrial dynamics [27]. Its misregulation has implications in the development of muscular atrophy [28]. The existence of a crosstalk between autophagy and the mitochondrial dynamics machinery, as well as with the apoptotic process, opens new questions and is in need of further investigations.

To reinforce the idea of an intimate connection between mitochondrial dynamics and function, recently published data have revealed that *in vivo* genetic ablation of fusion (*Opa1* knock-out mice [29, 30], *Mfn1/Mfn2* double knock-out mice [5]), or fission (*Drp1* knock-out mice [31]) proteins results in early embryonic lethality. Other data reveal mutations or abnormal regulation of mitochondria shaping proteins in many pathological conditions, as we will see below.

## 2. Cancer

According to the classification of the hallmarks of cancer by Hanahan and Weinberg [32], a cell needs a multistep process to become tumoral and, later on, to develop metastasis. Mitochondria are crucially positioned for establishing resistance to cell death and sustaining proliferative signallings. Their role is essential for the metabolic shift to glycolysis (the so-called Warburg effect), common in tumoral cells. Increasing evidence shows the involvement of mitochondrial dynamics in cancer development (see Table 1).

### 2.1. Escaping Cell Death and Regulating Mitochondrial Morphology: A Role for the Bcl-2 Family Proteins

Escaping death signals is one of the first characteristics of a tumoral cell. Bcl-2 family proteins play an important role in balancing life and death signals [33] converging on mitochondria and, at the same time, in regulating changes in mitochondrial morphology. Generally, prosurvival signals are associated with elongated mitochondria, while cell death is usually accompanied by mitochondrial fragmentation. BCL-2 is a tumoral marker overexpressed in many lymphomas contributing to resistance to cell death [34–36]. CED-9, the homolog of BCL-2 in *C. elegans*, is able to interact with MFN2-inducing mitochondrial fusion [37]. BCL-X_L_ promotes instead fission stimulating the DRP1 GTPase activity [38]. The proapoptotic BAK and BAX stabilize DRP1 on mitochondria promoting fission [39], indeed, *Bak* ^−/−^ *Bax* ^−/−^ cells have an elongated mitochondrial network [40]. Interestingly, BAX colocalizes with DRP1 and MFN2 at sites of fission so promoting mitochondrial membrane permeabilization [39]. Finally, consistent with their proapoptotic role, NOXA and PUMA trigger DRP1-dependent mitochondrial fragmentation [41]. 

### 2.2. Metabolic State and Mitochondrial Shape Changes

Another feature of tumoral cells is the already mentioned shift from the production of ATP by oxidative phosphorylation to a glycolytic phenotype despite the presence of oxygen [32]. It is not yet clear if this metabolic modification is an adaptation to a hypoxic microenvironment or the result of defects in OXPHOS respiration. Nevertheless, the existence of a double relationship between mitochondrial morphology and metabolic state is, however, increasingly evident. In OXPHOS cells, mitochondria appear elongated (State III); in glycolytic cells, they have a more fragmented phenotype (state IV) [46, 47]. The molecular mechanism underlying this phenomenon involves fusion proteins: reduced levels of MFN2, MFN1, or OPA1 results in the inhibition of TCA cycle, the decrease of oxidative phosphorylation, and the increase of glycolysis and lactic fermentation [42, 43]. Moreover, in a tumoral mass, the cellular response to hypoxia triggers mitochondrial elongation, dependent on HIF1; this, in turn, increases the resistance to apoptotic stimuli [48]. On the other hand, an efficient oxidative phosphorylation, and the consequent optimal mitochondrial membrane potential, (Δ*ψ*), is necessary for mitochondrial fusion [49, 50].

### 2.3. Cell-Cycle Regulation: When Cell Division Means Mitochondrial Fragmentation

It has been shown that mitochondria undergo fragmentation during the S and M phase of the cell cycle to allow a limitation of the mutation rate during DNA replication, through a temporary decrease of oxidative phosphorylation (which is the main source of ROS). Such fragmentation is also necessary for an equal segregation of mitochondria between the daughter cells [51, 52]. This process is mainly regulated by CDK1/Cyclin B complex, which phosphorylates DRP1 at the beginning of the S phase, resulting in DRP1 recruitment on mitochondria and subsequent mitochondrial fragmentation [53]. Thus, in normal conditions, the fragmentation of mitochondria, required during cell division, is a DRP1- and CDK1-dependent process, but in many tumors the cell cycle is dysregulated, and CDK1 activity becomes altered. It remains to be investigated whether this has an effect also on the morphology of mitochondria.

### 2.4. ROS Production and Mitochondrial Fragmentation

To continue this overview, ROS can be considered as both initiator factors of the tumor (inducing genome and mtDNA mutations) and enhancing factors giving a higher rate of proliferation to the cells [54, 55]. ROS production is mainly attributed to mitochondria, at the level of respiratory chain, and, in case of mitochondrial fragmentation, it is enhanced. Significantly, ionizing radiation is accompanied by ROS production and mitochondrial fragmentation in a DRP1-dependent way, so contributing to genome instability and carcinogenesis [44].

Interestingly, clinical studies of lung adenocarcinoma reveal a role for DRP1, independent of the mitochondrial morphology. In this tumor, DRP1 is overexpressed, but is sequestered in the nucleus by hHR23A, so avoiding its localization on mitochondria and conferring resistance to cisplatin [56]. A central role for mitochondrial dynamics also emerges in other studies. IL-6 dependent cancer cachexia is characterized by MFNs mRNA reduction and FIS1 mRNA upregulation [57]. FIS1 is also upregulated in some subtypes of human malignant melanoma [58].

In a more general way, cancer cells share characteristics with stem cells, in particular regarding mitochondrial morphology, localization, function, and mtDNA content [59]. Of note, in embryonic stem cells, there is a growth factor *er*v-1-like (GERF)-dependent DRP1 downregulation, which leads to mitochondrial elongation and an enhanced cell viability [60].

Finally, in lymphocytes mitochondria fission and relocalization to the uropod are necessary for the polarization and chemotaxis of these cells [45], so unravelling a possible role for mitochondrial morphology also in metastasis formation, where the acquisition of migratory capability represents the main feature of a metastatic cell phenotype.

## 3. Neurodegenerative Diseases

### 3.1. Beyond the Morphology: Physiological Mechanisms Affected by Altered Mitochondrial Dynamics in Neurons

Before starting the examination of different pathologies directly related to mitochondrial dynamics failure or imbalance (see Table 2), we would like to give an overview of some of the possible mechanisms whereby mitochondrial dynamics alterations can lead to neurodegeneration: aberrant mitochondrial trafficking, altered interorganellar communication and impaired mitochondrial quality control [61].

#### 3.1.1. Mitochondrial Trafficking

Neurons, especially motor neurons, are characterized by long axons up to more than one meter at the end of which synapses exert their role in cellular-cellular communication. The resulting importance of mitochondrial anterograde transports to the synapses (to ensure ATP production necessary for neurotransmitters vesicles to be discarded [62]) and of retrograde movement to the soma of the cell are both clearly evident. Mitochondria rely on dynein/dynactin motor for the anterograde movement, on kinesin motor for the retrograde one, and on MIRO and MILTON as an additional mitochondrial linker and regulatory proteins [63]. Thus, a defect in the cellular motors, or in the mitochondria compartment to be loaded as cargo, could result in a mitochondrial deficit at synapses and in neurodegeneration. There is no direct correlation described so far in patients although, in some sporadic cases of Alzheimer disease (AD), trafficking alteration has been observed due to mutation in *Kinesin1* [64]. That said, increasing data are emerging in experimental models. Anterograde and retrograde trafficking is altered in Amyotrophic lateral sclerosis (ALS) mouse models in which SOD1 [65, 66], guanin-nucleotide exchange factor (GEF) and TAR DNA-binding protein 43 (TDP-43) are mutated [67, 68]. Noteworthy, a role for mitochondrial trafficking impairment has been demonstrated in pathologies not only affecting long axon neurons but also short cortex and hippocampal ones (this is the case of Alzheimer disease—AD—models) [64, 69, 70]. Similar observations come from works in a Huntington's disease (HD) mouse model, in which mutated *Htt* (the gene of HUNTINGTIN protein) is able to block mitochondrial movement [71] and causes a redistribution of kinesin and dynein in primary cortical neurons [72]; in Parkinson disease (PD) cellular and mouse models where PINK1 has been shown to interact with MIRO and MILTON [73], as well as with *α*-SYNUCLEIN, LRRK2, and PARKIN, to disrupt the microtubule network in the cell [74–76]. 

#### 3.1.2. Mitochondria-Associated Membranes and Ca^2+^ Homeostasis

In recent decades, a functional role for mitochondria-ER interactions and Ca^2+^-signalling implications has emerged [6]. The sites where these two organelles interact are defined as mitochondria-associated membranes (or MAMs), and MFN2 activity is pivotal in ER-mitochondria tethering and MAM formation. MAMs have a role in regulating calcium crosstalk between ER and mitochondria, so avoiding Ca^2+^ overload in mitochondria in physiological condition and revealing, thus, an unexpected connection with mitochondrial trafficking. In fact, MIRO is a calcium-binding protein; it has been proposed that only in calcium unbound state (low local Ca^2+^ concentration) is it able to interact with MILTON, so allowing movement of mitochondria [101]. Thus, an alteration in Ca^2+^ homeostasis, due to abnormal ER-Mito crosstalk, results in impaired mitochondrial movement and consequently in neurodegeneration [101]. Moreover, Amyloid *β*, a constituent of extracellular neurite plaques in AD, is abundant in MAMs, contributing to interorganellar dysfunctions [102]. Altered MAM organization has been proposed also for spinocerebellar ataxias (SCA), due to mutation in the regulatory subunit of Protein phosphatase 2A, PPPR2B [103], and for PD, due to mutation in the subunit 2b of phospholipase A2 (iPLAS2b). The latter is important in ER-mitochondria crosstalk during apoptosis mediated by ER stress [104].

#### 3.1.3. Mitochondrial Quality Control

The role of mitochondrial quality control is becoming increasingly prominent in the explanation of neurodegenerative diseases such as Parkinson disease (PD) and others. A general mechanism of mitochondrial quality control relies on the PINK1/PARKIN pathway, deeply studied *in vitro*. Briefly, loss of Δ*ψ* induces stabilization of PINK1 on the OMM and allows PARKIN recruitment on mitochondria. This, in turn, leads to ubiquitination of mitochondrial substrates and their interaction with p62 and LC3 so as to induce the engulfment of mitochondria inside the autophagosome [24, 25]. MFNs, for example, are ubiquitinated in a PARKIN-dependent manner [105] and then degraded by proteasome [106]. Others showed that DRP1 stability is also regulated by PARKIN [107].

### 3.2. Focus on the Pathologies

Coming back to the pathologies, in this paragraph, we will focus on the links between some of them and the mitochondrial dynamics.

#### 3.2.1. Alzheimer Disease

The main clinical feature of Alzheimer disease (AD) is the accumulation of extracellular deposits of amyloid *β* (A*β*) plaques and tau-containing intracellular neurofibrillary tangles in the brain, these leading to progressive neuronal death. From a morphological point of view, neurons expressing amyloid protein precursor (APP), or Amyloid *β*, show abnormal levels of mitochondrial shaping proteins with downregulation of MFNs and OPA1 and upregulation of DRP1 and FIS1 [69, 70, 108, 109]. Amyloid *β* interacts with DRP1 [77], promoting mitochondrial fission in a DRP1 S-nitrosilation-dependent manner [110, 111]. Tissues from patients affected by AD show mitochondria with disrupted cristae structure [112] and reduction of the number of mitochondria in dendrites [69]. Interestingly, although cell-cycle-coupled events are rare in postmitotic cells, the activity of CDK1 and CDK5 is enhanced in AD. CDK5 phosphorylates tau [78], while a high level of phosphorylated DRP1 at Serine 616 appears to be dependent on both CDK1 and protein kinase C *δ* (PKC *δ*) [79], as it has been shown in rat primary neurons.

#### 3.2.2. Huntington's Disease

A mitochondrial connection is emerging also in Huntington's disease (HD); mitochondrial succinate dehydrogenase (SDH, complex II), aconitase defects [80], and mtDNA damage [113] have been reported in *in vivo* models of HD. In addition, 3-nitropropionic acid, an irreversible inhibitor of complex II, has been shown to induce mitochondrial fragmentation and HD-like symptoms in rats and mice [81]. Of note is that primary striatal neurons from HD mouse models reveal mitochondrial fragmentation [114] with an alteration of mitochondrial shaping proteins in the brain (DRP1 and FIS1 upregulation, OPA1 and MFN1 downregulation) [115]. Mutant HUNTINGTIN is able to bind DRP1, increasing its GTPase activity and inducing mitochondria fragmentation both in mice and in human brains [82]. This phenotype is rescued by MFNs or DRP1-K38A-dominant negative overexpression and by the use of two DRP1 inhibitors, mdivi1 or miR-499 [116–120].

#### 3.2.3. Parkinson Disease

Independent studies identified **α*-synuclein*, *Pink1*, *Parkin*, *DJ-1,* and *Leucine-rich repeat kinase 2* (*LRRK2*) as commonly mutated genes in Parkinsonism. *α*-SYNUCLEIN and LRRK2 have been proposed as playing a role in microtubule organization and, thus, in mitochondrial trafficking [74, 75]. PINK1 and PARKIN are key proteins in mitochondrial quality control [24, 25], as we discussed above. Interestingly, opposite to what has been observed in drosophila [121, 122], downregulation of PINK1 or PARKIN by siRNA in neuroblastoma cells leads to mitochondrial fragmentation [83]. Also in this case, this fragmentation is rescued by genetically forcing the mitochondria morphology equilibrium towards fusion, or by treatment with the calcineurin inhibitor FK506 [84, 85]. The recruitment of PARKIN to mitochondria has been nicely investigated in different models; generally, it has been shown that upon mitochondrial membrane depolarization PARKIN is recruited to mitochondria both in primary and cultured cell models [25], but this mechanism is also inhibited in primary neurons [86]. The apparent discrepancy in the results among different experimental models could be explained, at least in part, by the observation that those cell models rely on different bioenergetic systems. It has been shown, in fact, that, in primary rat neurons, which largely depend on mitochondrial respiration to produce ATP, or in nonneuronal cells forced to mitochondrial respiration, PARKIN fails to translocate to mitochondria after membrane depolarization in contrast to what is observed in cells relying on glycolytic production of ATP [86]. This suggests that additional regulatory and/or protective mechanisms against mitochondrial damage have to be investigated in neurons. We should also consider that, in all these studies, loss of Δ*ψ* is obtained by treating cells with high concentrations (or for long terms) of the protonophores CCCP or FCCP. A common challenge in the next future will be the identification and the use of more physiological stimuli to induce mitochondrial damage, and mitophagy, to better mimic the *in vivo* mechanism of pathophysiology of neurodegenerative diseases. By this way, it will be possible to clarify some of the discrepancies remaining in the field such as, for example, the differences observed so far for the PINK1/PARKIN functional interaction in various cell lines; or considering the loss of Δ*ψ* (artificially induced and forced in all the *in vitro* experiments performed until now) as the only event triggering mitophagy, another point highly debated and controversial to date.

A mouse model of *DJ-1* knockout presents mitochondrial fragmentation [123], increased ROS production, and reduced respiration rates accompanied by basal autophagy impairment [124]. Complex I dysfunctions are common in PD, and its inhibition, by rotenone or 6-hydroxydopamine (6-OHDA) treatment, results in a DRP1-dependent mitochondrial fragmentation in neurons [125, 126], so suggesting another link between bioenergetic dysfunctions and altered mitochondrial dynamics in neurodegenerative diseases.

#### 3.2.4. Amyotrophic Lateral Sclerosis

In the 20% of patients affected by an autosomal dominant form of amyotrophic lateral sclerosis (ALS), a gain of function mutation in SOD1 has been detected [87]. Defects of complex IV activity and mtDNA rearrangement have been also reported in patients affected by ALS [127, 128]. Abnormal aggregation of mitochondria is common in the subsarcolemmal region of muscles and in the anterior horn neurons of the lumbar spinal cord [88, 89]. At ultrastructural levels, in the case of ALS, mitochondria show disorganized cristae with expansion of intermembrane space (IMS) [90, 91]. Of note, the overexpression of a mutated form of SOD1 in ALS (SOD1-G93A) induces fragmentation of mitochondria in NSC-34 motoneuronal-like cells [92]. Moreover, motor neurons from mice overexpressing SOD1-G93A show impaired mitochondrial fusion both in axons and in the cell body with impaired retrograde axonal transport and reduction of frequency and speed of the movement [93].

#### 3.2.5. Autosomal Dominant Optic Atrophy

*Opa1* mutations are responsible for the autosomal dominant optic atrophy (ADOA) [94, 95]. Contrary effects regarding oxidative phosphorylation impairment related to this disease have been published. Phosphorus magnetic resonance spectroscopy and luciferin/luciferase assays of muscle cells from ADOA patients showed a reduced ATP production mainly due to reduced complex I activity [129, 130]. On the other hand, in other studies, no energetic impairment was observed in lymphoblasts and muscle cells from ADOA patients [96, 97]. Whatever the case, consistent with the role of OPA1 in regulating mitochondrial dynamics, mitochondrial fragmentation is a common feature of ADOA with a severity score of the pathology directly proportional to the level of fragmentation observed [97, 98, 130]. Interestingly, fibroblasts from ADOA patients also reveal a major sensitivity to death stimuli [98], in line with the antiapoptotic role of OPA1 described above [8].

#### 3.2.6. Other Neuropathies and Neurological Disorders

Among other neuropathies Leber hereditary optic neuropathy (LHON) has, as a primary cause, mutations in mtDNA [131]. Recently, a possible link with mitochondrial dynamics has been presented in a genome-wide linkage scan study, in which a mutation in *Parl* (the mitochondrial protease responsible for OPA1 cleavage, [132]) has been associated with LHON [133].

MFN2 is involved in the most common form of the autosomal dominant axonal Charcot-Marie-Tooth (CMT2A) disease [99]. Moreover, ganglioside-induced differentiation-associated protein 1 (GDAP1), whose mutations cause an autosomal recessive form of CMT, appears to be related to mitochondrial fission in mammalian cells [100].

In addition, in a mouse model of apoptosis-inducing factor (AIF) deficiency, MFN1 levels are decreased in the cerebellum and are accompanied by death of Purkinije cells [134]. This phenomenon is generally observed in many neurological diseases such as autism, HD, AD, multiple system atrophy, and epilepsy [135]. Consistently, also in *Mfn2* knock-out systems, death of Purkinije cells has been observed, confirming a role for MFNs in protecting against lack of mtDNA and dysfunction of mitochondria in the cerebellum [136].

In the last decade, a link between neurological and lymphatic aspects has emerged in schizophrenia [137]. Recently, a study in schizophrenia-derived lymphoblastoids revealed altered oxidative phosphorylation at level of complex I and clustering of mitochondria in a limited area of the cell, with a reduction in OPA1 expression levels [138].

To conclude, neurons rely on mitochondrial distribution, function, and dynamics to allow synapses and dendrites formation, energy supply, and quality control. The main properties of neurons constitute risk factors themselves if we consider the effects of mitochondrial dysfunctions. First, they are cells highly demanded in energy; second, they have long processes connecting the soma to synapses and dendritic spines; third, they are long-lived postmitotic cells. Although knowledge is increasing about mitochondrial role in neurodegenerative diseases, much remains to be elucidated, in particular, why different subpopulations of neurons are more vulnerable to mitochondrial fusion/fission imbalance and dysfunctions. 

## 4. Neuroinflammatory and Autoimmune Diseases

Little is known about the link between mitochondrial dynamics and neuroinflammatory or autoimmune diseases. In this section we present correlations described to date with multiple sclerosis (MS) and type I diabetes, as examples for this category of diseases (see Table 3). We then introduce some general outcomes about mitochondrial dynamics and T-cell compartment with the potential for opening up new perspectives regarding cellular mechanisms and clinical therapies for many pathologies.

### 4.1. Optic Neuritis and Multiple Sclerosis

Optic neuritis (ON) is a neuropathy characterized by demyelination of the optic nerve; it can be present by itself or as part of MS. Multiple sclerosis is an autoimmune disorder characterized by chronic demyelination of the central nervous system (CNS). The pathogenesis of MS is thought to involve self-antigen-reactive T lymphocytes that have the capacity to invade the CNS and to promote tissue damage.

It is not rare to find mtDNA mutations and mitochondrial abnormalities in patients affected by ON [143]. For instance, in a case of *OPA1* mutation (S646L) ADOA has been shown to be associated with MS. This mutation leads to reduction of respiratory rates with lower ATP production [139], which is implicated in demyelination of axons in MS [140]. Interestingly, it has been shown that symptoms of autoimmune disorders, including MS, improve during pregnancy due, at least in part, to the expression of embryo-derived preimplantation factor (PIF). This protein is able to reduce neuroinflammation and to promote neural repair in the experimental autoimmune encephalomyelitis (EAE) model of MS, through a general decrease in proinflammatory cytokine and chemokine secretion, and a downregulation of proapoptotic factors and of activating and migrating proteins such as OPA1 [144].

### 4.2. Type I Diabetes

Type I diabetes is an autoimmune disorder caused by autoimmune elimination of insulin-producing *β* cells in the pancreas, clinically leading to increased glucose in blood and urine. Coronary endothelial cells from diabetic mice are characterized by fragmented mitochondria with a downregulation of OPA1 and an upregulation of DRP1 [141]. In addition, Prohibitin (Phb) has been shown to have a protective role in *β* cells [142]. Interestingly, *phb*-genetic ablation results in aberrant mitochondrial cristae structure and an increased apoptosis, dependent on increased proteolytic processing of OPA1 [15]. Moreover, it has been shown that the embryo-derived preimplantation factor (PIF) also prevents type I diabetes in mouse models of this disease [145].

### 4.3. Mitochondrial Morphology and T-Cell Function

The importance of mitochondrial localization and activity in T cell function is well established. T cells are activated at the so-called “immunological synapse” between a T cell and an antigen-presenting cell (APC) [146]. Mitochondria usually fragment and relocalize at the immunological synapse in close proximity to the plasma membrane to buffer Ca^2+^ entrance and to avoid calcium-dependent T-cell inactivation [147]. Moreover, upon activation, T cells migrate to the site of inflammation towards a chemoattractant gradient. Our group reported that mitochondria allow lymphocyte migration by relocating and accumulating at the uropod (the posterior area of an activated T cell) where they can provide the necessary energy to class II myosin proteins, these being the major cellular motors. Interestingly, this reorganization needs mitochondrial fission while a forced fusion inhibits both mitochondrial relocalization and lymphocyte migration [45]. In recent years, the polarization of mitochondria towards cell-cell surface has also been shown to occur between natural killer (NK) and tumoral cells [148, 149].

Mitochondrial fission, driven by FIS1 and DRP1, also contributes to the immune system tolerance and the tumor-immune escape in a galectin-1 (GAL-1)-dependent manner. GAL-1 sensitizes resting and activated T cells to FAS-mediated cell death program, which is characterized by mitochondrial dysfunctions, membrane potential alteration, mitochondrial fission, and cytochrome *c* release [150].

It still remains to be elucidated whether or not mitochondrial dynamics play a crucial role in other important physiological processes in T cells.

Finally, CD47 can trigger cell death in a B lymphocyte leukemia. Also this apoptotic pathway is characterized by DRP1 translocation to mitochondria, which depends on chymotrypsin-like proteases, Δ*ψ* loss and ROS production [151]. This last observation is an indication of a possible role of mitochondrial dynamics in general in the whole immune system, more than only in the T-cell physiology and pathophysiology.

## 5. Conclusion

The observations here presented suggest a prominent role for mitochondrial dynamics in a plethora of pathways from cell proliferation to resistance to apoptosis, from cell-energetic requirements to quality control mechanisms, from homeostasis to activation and movement of immune system cells. However our observations also suggest the potential for many new findings to come. The aim of future studies would, therefore, be to extend our knowledge of basic mechanisms underlying pathologies, and their relationship with mitochondrial morphology alterations. This, in turn, could make it possible to draw up novel strategies and treatments to improve the prognosis of an increasing number of patients.

## Figures and Tables

**Table 1:** Mitochondrial dynamics and cancer.

**Pathology**	**Proteins involved (expression level)**	**Mitochondrial phenotype**	**Mechanisms of pathophysiology involving mitochondria**
**Different types of tumors**	MFN1, MFN2, OPA1 ↓↓ DRP1, FIS1 ↑↑	Fragmentation.	Inhibition of TCA cycle and oxidative phosphorylation, mitochondrial membrane permeabilization; fission accompanied by ROS production, polarization and chemotaxis of lymphocytes and tumoral cells [42–45].

**Table 2:** Mitochondrial dynamics and neurodegenerative diseases.

**Pathology**	**Proteins involved ** **(expression level and/or mutation)**	**Mitochondrial phenotype**	**Mechanisms of pathophysiology involving mitochondria**
**Alzheimer disease**	MFN1, MFN2, OPA1 ↓↓ DRP1, FIS1 ↑↑ KINESIN mutation	Fragmentation, disruption of cristae structure, reduction in number of mitochondria in dendrites, impaired mitochondrial trafficking, defects in KGDH complex, PDH complex and COX.	*β* amiloyd accumulation and interaction with DRP1, enhanced CDK1 activity, altered interaction between mitochondria and Kinesin motor complex in cerebral cortex [77–79].

**Huntington's disease**	MFN1, MFN2, OPA1 ↓↓ DRP1, FIS1 ↑↑ HTT mutation	Fragmentation; impaired mitochondrial trafficking, defects in SDH (complex II) and Aconitase.	HTT interaction with DRP1, increased calcineurin and DRP1 activity, redistribution of kinesin and dynein motor complexes in striatal neurons [80–82].

**Parkinson disease**	Parkin mutation or ↓↓ Pink1 mutation or ↓↓ DJ-1 mutation DRP1 ↓↓ MFN2 *α*-synuclein mutation LRRK2 mutation	Fragmentation, impaired mitochondrial trafficking.	Altered interaction between mitochondria and motor complexes, impaired mitophagy of damaged mitochondria in *substantia nigra *[74, 75, 83–86].

**Amiotrophic lateral sclerosis**	SOD mutation GEFmutation TDP-43 mutation	Fragmentation, disruption of cristae structure with expansion of IMS, impaired mitochondrial trafficking, complex I dysfunctions.	Toxicity associated to the formation of aggregates of mutant SOD, in subsarcolemmal region of muscles and anterior horn neurons of lumbar spinal cord [87–93].

**Autosomal dominant optic atrophy**	OPA1 mutation	Fragmentation, complex I dysfunctions.	Major sensitivity to death stimuli in retinal ganglion cells and optic nerve [94–98].

**Charcot Marie Tooth Type 2**	MFN1 mutation GDAP1mutation	Fragmentation (MFN1 mut) or elongation (GDAP1 mut).	MFN1: probably alteration in ER-mitocondria tethering and Calcium signalling [99]; GDAP1: altered localization of GDAP1 [100].

**Table 3:** Mitochondrial dynamics and neuroinflammatory and autoimmune diseases.

**Pathology**	**Proteins involved ** **(expression level and/or mutation)**	**Mitochondrial phenotype**	**Mechanisms of pathophysiology involving mitochondria**
**Multiple sclerosis**	OPA1 mutation	Fragmentation.	Reduction of respiratory rates with lower ATP production [139, 140].

**Type 1 diabetes **	OPA1 ↓↓ DRP1 ↑↑ PHBs mutation	Fragmentation, disruption of cristae structure.	Alteration in OPA1 processing in *β*-cells in the pancreas and coronary endothelial cells from diabetic animals [141, 142].
